# Peroxide of Hydrogen

**Published:** 1887-07

**Authors:** 


### Peroxide of Hydrogen

Is recommended by Dr. B. W. Richardson, in the *Asclepiad*, as a remedy of some value in whooping cough. He believes it to act like dilute nitric acid, subduing the spasmodic paroxysm, checking secretion, and shortening duration of the disease. It is, however, more effective than this acid. The formula employed is—hydrogen peroxide ʒvj., glycerine fʒiv., water to f℥iij. Of this mixture half a fluid ounce is given in a wine-glass-full of water five or six times a day.

